# Health disparities in past influenza pandemics: A scoping review of the literature

**DOI:** 10.1016/j.ssmph.2022.101314

**Published:** 2022-12-09

**Authors:** Angela D'Adamo, Alina Schnake-Mahl, Pricila H. Mullachery, Mariana Lazo, Ana V. Diez Roux, Usama Bilal

**Affiliations:** a Edward J. Bloustein School of Planning and Public Policy, Rutgers, The State University of New Jersey, New Brunswick, NJ, USA; b Department of Epidemiology, Johns Hopkins Bloomberg School of Public Health, Baltimore, MD, USA; c Urban Health Collaborative, Drexel Dornsife School of Public Health, Philadelphia, PA, USA; d Department of Health Management and Policy, Drexel Dornsife School of Public Health, Philadelphia, PA, USA; e Department of Community Health and Prevention, Drexel Dornsife School of Public Health, Philadelphia, PA, USA; f Department of Epidemiology and Biostatistics, Drexel Dornsife School of Public Health, Philadelphia, PA, USA; g Department of Health Services Administration and Policy, Temple University College of Public Health, Philadelpha, PA, USA

**Keywords:** Health inequities, Influenza, Pandemic, Social class, Ethnic groups

## Abstract

**Objective:**

The COVID-19 pandemic has exacerbated existing health disparities. To provide a historical perspective on health disparities for pandemic acute respiratory viruses, we conducted a scoping review of the public health literature of health disparities in influenza outcomes during the 1918, 1957, 1968, and 2009 influenza pandemics.

**Methods:**

We searched for articles examining socioeconomic or racial/ethnic disparities in any population, examining any influenza-related outcome (e.g., incidence, hospitalizations, mortality), during the 1918, 1957, 1968, and 2009 influenza pandemics. We conducted a structured search of English-written articles in PubMed supplemented by a snowball of articles meeting inclusion criteria.

**Results:**

A total of 29 articles met inclusion criteria, all but one focusing exclusively on the 1918 or 2009 pandemics. Individuals of low socioeconomic status, or living in low socioeconomic status areas, experienced higher incidence, hospitalizations, and mortality in the 1918 and 2009 pandemics. There were conflicting results regarding racial/ethnic disparities during the 1918 pandemic, with differences in magnitude and direction by outcome, potentially due to issues in data quality by race/ethnicity. Racial/ethnic minorities had generally higher incidence, mortality, and hospitalization rates in the 1957 and 2009 pandemics.

**Conclusion:**

Individuals of low socioeconomic status and racial/ethnic minorities have historically experienced worse influenza outcomes during pandemics. These historical patterns can inform current research to understand disparities in the ongoing COVID-19 pandemic and future pandemics.

## 1 Introduction

COVID-19 caused more than 18 million deaths worldwide in 2020 and 2021 (Johns Hopkins University of Medicine Coronavirus Resource Center; Wang, Paulson, & Pease, 2022). There have been wide spatial, socioeconomic, and racial/ethnic disparities in COVID-19 incidence, hospitalizations, and mortality during the COVID-19 pandemic (Bassett et al., 2020; Bilal et al., 2021; American Public Media Research Lab Staff; Chen & Krieger, 2020; Mackey, Ayers, Kondo, & et al., 2020; Riou et al., 2021). Although caused by different pathogens, COVID-19 and influenza are both contagious respiratory illnesses caused by viruses, transmitted by respiratory droplets and aerosols (Cowling et al., 2013), and share symptoms including fever, chills, headache, myalgias, cough, fatigue, and more severe manifestations leading to hospitalization and mortality (Solomon et al., 2020). Though influenza has killed 12,000 to 61,000 people annually in the US since 2010 (Centers for Disease Control and Prevention, 2020), influenza virulence and infectivity changes frequently, leading to annual differences in transmission and mortality. Influenza pandemics emerge when a large mutation in the virus occurs, leading to a strain for which there is little to no existing immunity (Kilbourne, 2006). With a lack of immunity, new virus strains can easily infect humans, efficiently transmit to other people, and because of these features, have the potential to cause pandemics.

According to the Centers for Disease Control and Prevention (CDC), there have been, so far, four confirmed influenza pandemics: 1918, 1957–1958, 1968, and 2009 (Monto & Fukuda, 2020; Centers for Disease Control and Prevention). These four remain the only pandemics with molecular confirmation of influenza virus spread. Controversy remains about the origin of previous pandemics, such as the 1889 “Russian flu” (Valleron et al., 2010), which despite its name, may have been caused by coronaviruses (Brüssow & Brüssow, 2021; Vijgen et al., 2005), or whether other epidemics, such as the 1977 one, reached pandemic status (Kilbourne, 2006; Monto & Fukuda, 2020). The 1918 influenza pandemic resulted from an H1N1 influenza A virus. The pandemic featured high mortality among otherwise healthy young adults (ages 20 to 39), and a large proportion of influenza-related deaths were associated with secondary bacterial infections (Monto & Fukuda, 2020). An estimated one-third of the world's population became infected with the virus and at least 50 million died globally (Centers for Disease Control and Prevention). The influenza pandemics of 1957–58 (henceforth referred as 1957) and 1968 originated in Hong Kong, involved the H2N2 and H3N2 influenza A strains, respectively (Monto & Fukuda, 2020), and each caused around 1 million deaths (Centers for Disease Control and Prevention; Centers for Disease Control and Prevention). Both strains caused high infection and mortality rates among the very young and very old (Monto & Fukuda, 2020). The 2009 pandemic was caused by an H1N1 influenza A virus that originated in Mexico (Monto & Fukuda, 2020). Adults older than 65 experienced lower morbidity and mortality than during usual flu seasons, while children and young and middle aged adults accounted for the majority of deaths, hospitalizations, and infections (Centers for Disease Control and Prevention, 2009). During the first year of infection, an estimated 151,700 to 575,400 people died worldwide (Centers for Disease Control and Prevention, 2009).

Prior reviews have summarized a single dimension of health disparities within pandemics, especially during the 1918 and 2009 pandemics (Mamelund, 2017; Roberts & Tehrani, 2020; Økland & Mamelund, 2019); in this paper, we scope the public health literature examining health disparities across multiple dimensions, including geographic, socioeconomic, and racial/ethnic disparities, during the 1918, 1957, 1968, and 2009 influenza pandemics, worldwide. Health disparities are unjust and preventable differences in health based on place or social group (e.g. race-ethnicity, socioeconomic status (SES), immigration status, or other social status) (Braveman, 2014). The examination of documented health disparities in previous influenza pandemics can help us better understand patterns and predictors of disparities in past pandemics and inform the understanding of disparities during the COVID-19 and future pandemics.

## 2 Methods

### 2.1 Search strategy

We conducted a scoping review (Peters et al., 2015) of the public health literature assessing health disparities during the 1918, 1957, 1968, and 2009 influenza pandemics. We selected scoping reviews as we anticipated highly heterogenous nature of the evidence, not amenable to a more quantitatively focus systematic review of the evidence. Scoping reviews aid summarizing complex research findings and identifying research gaps (Peters et al., 2015). We employed the PICOT (population, intervention/exposure, comparison, outcome, and time) framework to guide inclusion criteria (Schardt et al., 2007). We did not limit the review to studies examining specific populations. We focused on disparities by individual and contextual SES (i.e., individual-level or neighborhood/area income, wealth, education, occupation, unemployment) and race/ethnicity. Comparison groups were either population subgroups (e.g., different racial/ethnic or SES groups), or geographic units. Outcomes of interest included any health-related outcome linked to influenza, including infection and severity (mortality, hospitalizations, etc.). We did not include studies that studied preventive interventions (e.g., vaccination, non-pharmaceutical interventions, etc.)

### 2.2 Identification and inclusion of articles

We included articles that met inclusion criteria within our PICOT framework, specifically: 1) publication in English, 2) assessment of short-term health disparities in any influenza-related health outcome, 3) set in one (or more) of the influenza pandemics of 1918, 1957, 1968, and 2009, and 4) empirical studies or reviews of empirical studies.

**Fig. 1:**
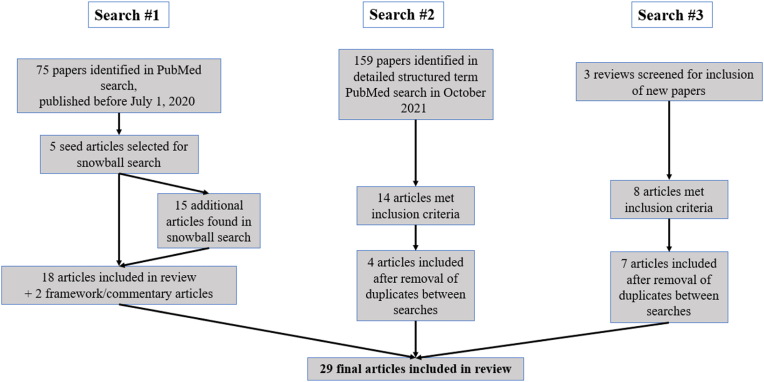
Search strategy.

We employed three complementary search strategies (Fig. 1). First, a snowball search that was initiated by searching in PubMed on July 1, 2020 using the following terms, without language or date restrictions: “influenza pandemics” AND “health disparities”. This resulted in 75 articles, 5 of which fit our PICOT criteria (Blumenshine et al., 2008; Dee et al., 2011; Grantz et al., 2016; Krishnan et al., 2020; Quinn et al., 2011) as screened by AD and UB. Using these “seed” articles, we then initiated a three step snowball search by: (1) reviewing the reference lists of included articles (backwards search); (2) looking for articles citing the included articles (forwards search), using Google Scholar; and (3) searching for articles concurrently cited with included articles (co-citation search) using Co-Cites (Janssens & Gwinn, 2015). We repeated the snowball search until further backwards, forwards, and co-citation searches did not render any more included articles. From the snowball search, 15 additional articles met inclusion criteria.

Second, in October 2021 we employed a more detailed structure term search in PubMed (((influenza OR flu) AND pandemic) AND (health AND (disparities OR inequalities OR inequities))), netting 159 articles which were screened for inclusion by AD and ASM. Third, we further reviewed three reviews of the literature that we found during the previous searches and that focused on specific disparities in specific influenza pandemics (Mamelund, 2017; Mamelund & Dimka, 2021; Økland & Mamelund, 2019), and screened their respective reference lists for inclusion of new papers.

These search strategies resulted in 18, 14, and 8 articles respectively, which following the removal of duplicates between searches resulted in 29 final included articles. Two of the five original “seed” articles were commentaries/frameworks (Blumenshine et al., 2008; Krishnan et al., 2020), which we excluded from the review, but retained for the snowball search (Fig. 1).

### 2.3 Data extraction

From each included article, one reviewer (AD) extracted information on publication date, geographic and temporal setting, health equity angle (SES or race/ethnicity), comparison groups, outcomes, measures of disparities, adjustment covariates, and main results. ASM and UB reviewed all extraction information and reconciled any differences in interpretation.

We categorized articles based on the comparison groups reported into articles looking at population subgroups (e.g., by SES or race/ethnicity) or articles comparing geographical units (e.g., neighborhoods, counties, etc.).

## 3 Results

In total, this review is based on 29 articles: 12 articles focused solely on the 1918 pandemic, 16 on only the 2009 pandemic, and one on the 1918, 1957, and 2009 pandemics. No articles examined the 1968 pandemic. Of the 29 articles, ten focused on socioeconomic disparities, thirteen on racial/ethnic disparities, and six examined both (see Table 1 for study characteristics and Table 2 for a summary of results; please note that a study may be included in multiple rows of Table 2 if it reported more than one type of disparity or result). The seventeen articles that examined outcomes with respect to socioeconomic factors examined nine different outcomes: hospitalizations (6), influenza/pneumonia mortality (6), excess mortality (2), incidence (4), severity of disease (2), and speed of transmission, ICU admissions, onset, and duration (1 each). Nineteen articles examined 11 different outcomes in relation to race or ethnicity: hospitalizations (7), influenza/pneumonia mortality (5), incidence (6), intensive care unit admissions (3), and risk of exposure, susceptibility, access to health care, pediatric deaths, onset, duration, and severity of disease, and characteristics of severe cases (1 each). Few studies reported disparities outside the US (10 studies out of 29). The data was very sparse for some racial/ethnic groups; few articles (5) examined American Indian/Alaska Native populations and only two looked at Native Hawaiian/Pacific Islanders. Most studies (21) used a relative measure of inequalities (hazard, rate, odds, prevalence, or probability ratios), while others (6) only compared rates and proportions. All studies but four adjusted for age and most (18) adjusted or stratified by sex or gender.

**Table 1:** Study characteristics for the 29 included articles.

Citation	Equity angle	Pandemic	Context	Unit of Analysis	Outcomes	Measure of Association	Covariates/Adjustments
Økland, 2019 (Økland & Mamelund, 2019)	Racial-ethnic	1918	US	a	a	a	a
Gamble, 2010 (Gamble, 2010)	Racial-ethnic	1918	US	a	a	a	a
Frankel, 1919 (Frankel & Dublin, 1919)	Racial-ethnic	1918	US	Individual	Influenza-pneumonia incidence, mortality	Comparison of rates	Age, sex, race/ethnicity
Brewer, 1918 (Brewer, 1918)	Racial-ethnic	1918	Army Camp (US)	Regiment	Influenza incidence	Comparison of rates	Number of weeks
Mamelund, 2006 (Mamelund, 2006)	SES	1918	City (Norway)	Individual	Influenza mortality	Hazard ratio	Age, gender, marital status, social class, apartment size
Mamelund, 2018 (Mamelund, 1918)	SES	1918	City (Norway)	Individual	ILI incidence	Probability differences, probability ratio	Gender, SES (number of rooms), pandemic wave
Sydenstricker, 1931 (Sydenstricker, 1931)	SES	1918	Nine cities in the US	Individual	Influenza mortality, incidence	Comparison of rates, rate ratio	Household size, crowding, sex, age
Bengtsson, 2018 (Bengtsson et al., 2018)	SES	1918	Norway	Individual	Excess influenza mortality	Comparison of rates, relative risk	Age, marital status, number of children, migrant status, urban status, and county of residence
Clay, 2019 (Clay et al., 2019)	SES	1918	US	Ecological (cities)	Excess mortality	Comparison of rates	Percentage of urban residents in 1910, proximity to WWI base, coal capacity
Wilson, 2018 (Wilson et al., 2018)	SES	1918	City (New Zealand)	Ecological (suburbs)	Influenza mortality	Rate ratio	NA
Grantz, 2016 (Grantz et al., 2016)	SES	1918	City (US)	Ecological (Census tracts)	Influenza & pneumonia mortality, transmissibility	Risk ratio	Population density, homeownership, unemployment, age
Britten, 1932 (Britten, 1932)	Racial-ethnic, SES	1918	Cities, County (US)	Individual	Influenza-pneumonia incidence; onset, duration, severity of disease; mortality	Comparison of rates	Age, sex
Rutter, 2012 (Rutter et al., 2012)	SES	2009	UK	Ecological (LSOAs)*	Influenza mortality	Rate ratio	Age, gender
Balter, 2010 (Balter et al., 2010)	SES	2009	City (US)	Ecological (neighborhoods)	Hospitalizations	Comparison of rates	Age
Lowcock, 2012 (Lowcock et al., 2012)	SES	2009	Province (Canada)	Individual	Hospitalizations	Comparison of proportions, odds ratio	Age, gender
Navaranjan, 2014 (Navaranjan et al., 2014)	Racial-ethnic	2009	Province (Canada)	Individual	Incidence	Odds ratio	Age, sex, children in household, material deprivation, chronic conditions, receipt of 2008 seasonal vaccine, tested prior to June 11th, 2009, length of stay in Canada, total deprivation, high proportion of neighborhood with low income, material deprivation, post-secondary education, chronic condition, residence in Toronto
Quinn, 2011 (Quinn et al., 2011)	Racial-ethnic	2009	US	Individual	Risk of exposure, susceptibility, access to care	Odds ratio	Age, gender, presence of children in household, income, education
Dee, 2011 (Dee et al., 2011)	Racial-ethnic	2009	US	Individual	ILI, hospitalization, pediatric deaths	Comparison of proportions	Age
Soyemi, 2014 (Soyemi et al., 2014)	Racial-ethnic	2009	State (US)	Individual	Hospitalizations, influenza mortality	Comparison of proportions, rate ratio	Age, gender
Truelove, 2011 (Truelove et al., 2011)	Racial-ethnic	2009	State (US)	Individual	Hospitalizations, mortality, ICU admissions	Rate ratio	Age, clinical condition, hospital course
Castrodale, 2009 (Castrodale, 2009)	Racial-ethnic	2009	States (US)	Individual	Influenza mortality	Comparison of rates, rate ratio	Age
Placzek, 2014 (Placzek & Madoff, 2014)	Racial-ethnic; SES	2009	State (US)	Individual	Influenza-related ICU stay	Comparison of rates, odds ratio	Age, gender, admission type, interactions between race and gender
Kumar, 2012 (Kumar et al., 2012)	Racial-ethnic; SES	2009	US	Individual	ILI incidence	Comparison of means, odds ratio	Income, education, age, gender
Nyland, 2015 (Nyland et al., 2015)	Racial-ethnic; SES	2009	UK	Individual	Clinical outcomes, primary healthcare access, secondary healthcare access, in-patient care	Odds ratio	Age, sex, English Index of Multiple Deprivation score, recorded obesity, smoking and chronic obstructive pulmonary disease, admission delay > or equal to 4 days and severity at presentation for admission
Mayoral, 2013 (Mayoral et al., 2013)	Racial-ethnic; SES	2009	Spain	Individual	Hospitalizations	Odds ratio	Age, gender, unfavorable medical factors, vaccination, type of encounter
Thompson, 2011 (Thompson et al., 2011)	Racial-ethnic; SES	2009	State (US)	Individual	Hospitalizations	Rate ratio, odds ratio, risk ratio	Age, gender, obesity status, high-risk medical conditions, antiviral treatment
Levy, 2013 (Levy et al., 2013)	Racial-ethnic; SES	2009	City (US)	Individual	Hospitalizations	Odds ratio	Age, access to care, medical conditions, area of residence
Esteban-Vasallo, 2012 (Esteban-Vasallo et al., 2012)	Racial-ethnic	2009	City (Spain)	Individual	H1N1 infection, severe cases (hospital admission, chronic pathology, pregnancy, delay in hospital admission, and ICU admission)	Comparison of rates, odds ratio, prevalence ratio	Age, chronic pathology, pregnancy, delay in hospital admission
Wilson, 2012 (Wilson et al., 2012)	Racial-ethnic	1918, 1957, 2009	New Zealand	Ecological (country)	Mortality, hospitalizations	Rate ratio	Age

a multiple units of analysis or outcomes. SES = socioeconomic status. US=United States. UK=United Kingdom. ILI=Influenza-Like Illness. ICU=Intensive Care Unit. LSOA = lower super-output areas.

**Table 2:** Summary of results by level/type of disparity and pandemic.

Main results	Influenza pandemic	Citation
Less affluent neighborhoods had worse outcomes compared to more affluent neighborhoods	1918	Clay, 2019 (Clay et al., 2019), Mamelund, 2006 (Mamelund, 2006), Grantz, 2016 (Grantz et al., 2016)
	2009	Rutter, 2012 (Rutter et al., 2012), Thompson, 2011 (Thompson et al., 2011), Levy (Levy et al., 2013), Balter, 2010 (Balter et al., 2010), Lowcock, 2012 (Lowcock et al., 2012)
Individuals of low SES had worse outcomes compared to individuals of high SES	1918	Sydenstricker, 1931 (Sydenstricker, 1931), Wilson, 2018 (Wilson et al., 2018), Mamelund, 2018 (Mamelund, 1918)
	2009	Lowcock, 2012 (Lowcock et al., 2012), Mayoral, 2013 (Mayoral et al., 2013), Levy, 2013 (Levy et al., 2013)
Racial/ethnic minority groups had worse outcomes than whites	1918	Økland, 2019 (Økland & Mamelund, 2019), Gamble, 2010 (Gamble, 2010), Frankel, 1919 (Frankel & Dublin, 1919)
	2009	Quinn, 2011 (Quinn et al., 2011), Dee, 2011 (Dee et al., 2011), Placzek, 2014 (Placzek & Madoff, 2014), Soyemi, 2014 (Soyemi et al., 2014), Mayoral, 2013 (Mayoral et al., 2013), Truelove, 2011 (Truelove et al., 2011), Thompson, 2011 (Thompson et al., 2011), Levy, 2013 (Levy et al., 2013), Navaranjan, 2014 (Navaranjan et al., 2014), Castrodale, 2009 (Castrodale, 2009), Brewer, 1918 (Brewer, 1918), Nyland, 2015 (Nyland et al., 2015), Esteban-Vasallo, 2012 (Esteban-Vasallo et al., 2012)
	1918, 1957, 2009	Wilson, 2012 (Wilson et al., 2012)
Racial/ethnic minority groups had increased risk of exposure compared to whites	2009	Kumar, 2012 (Kumar et al., 2012), Quinn, 2011 (Quinn et al., 2011)
Individuals of low SES/living in less affluent neighborhoods/racial/ethnic minorities had better outcomes	1918	Gamble, 2010 (Gamble, 2010), Grantz, 2016 (Grantz et al., 2016), Mamelund, 2018 (Mamelund, 1918), Frankel, 1919 (Frankel & Dublin, 1919), Britten, 1932, Brewer, 1918 (Brewer, 1918)
	2009	Placzek, 2014 (Placzek & Madoff, 2014), Nyland, 2015 (Nyland et al., 2015), Esteban-Vasallo, 2012 (Esteban-Vasallo et al., 2012)

**Footnote**: SES = socioeconomic status; a study may be included in multiple rows if it includes multiple results or disparities.

We found eight articles that assessed outcomes in the 1918 pandemic in relation to SES, specifically illiteracy, homeownership, occupation, apartment size, and unemployment (Clay et al., 2019; Grantz et al., 2016; Mamelund, 2006), two at the neighborhood level and six at the individual level. Grantz et al. looked at neighborhood-level factors in Chicago and found that cumulative incidence of influenza and pneumonia mortality was 34% and 20% lower per 10% higher census tract homeownership and unemployment rate (Grantz et al., 2016), respectively. Clay et al. examined socioeconomic factors across U.S. cities and found that, compared to cities with the lowest percentage of illiterate residents, cities with the highest percentage of illiterate residents had 21.3 additional excess deaths per 10,000 residents during the pandemic (Clay et al., 2019). Mamelund examined individual- and household-level socioeconomic factors in Norway and found that influenza mortality rates were 49% higher in individuals in the deprived social class compared to the class considered advantaged in terms of income, education, and employment (Mamelund, 2006). Additionally, Bengtsson et al. examined differences in excess mortality in Norway by individual-level social class and found that among occupational groups (white-collar, skilled, low-skilled, unskilled, farmers), low-skilled workers had the highest excess mortality rate and skilled workers had a significantly lower death rate than unskilled and low-skilled workers (Bengtsson et al., 2018).

We also found eight studies that examined socioeconomic factors in the 2009 pandemic (Balter et al., 2010; Kumar et al., 2012; Levy et al., 2013; Lowcock et al., 2012; Mayoral et al., 2013; Placzek & Madoff, 2014; Rutter et al., 2012; Thompson et al., 2011). Balter et al. reported hospitalization rates two times higher in high-poverty neighborhoods than in low-poverty neighborhoods in New York City (Balter et al., 2010). Similarly, Rutter et al., found that influenza mortality rates were three times higher in the least affluent compared to the most affluent areas of England (Rutter et al., 2012). One study examining neighborhood-level factors in Ontario found that during the first and second 2009 pandemic waves, hospitalized individuals were more likely to have a lower education level and live in deprived neighborhoods than those who were not hospitalized (high school or less; Odds Ratio (OR) = 2.28 and 1.77 in first and second pandemic phases, respectively) and live in deprived neighborhoods (OR = 3.46 and 1.54 in first and second phases, respectively) (Lowcock et al., 2012). Similarly, in a study examining the association between education level and hospitalizations in New York City, adults with no education beyond high school had 4.5 times higher hospitalization odds than adults with some college education, and those without a high school education had 32 times higher odds of hospitalization than those with some college education (Levy et al., 2013).

We found five articles exploring racial/ethnic disparities in the 1918 pandemic in the US (Brewer, 1918; Britten, 1932; Frankel & Dublin, 1919; Gamble, 2010; Økland & Mamelund, 2019). Økland reviewed military, survey, and insurance data in the US and found heterogeneous disparities. Black soldiers had 2.3 times lower influenza incidence than white soldiers (Økland & Mamelund, 2019), but higher excess mortality and case fatality than white soldiers (Økland & Mamelund, 2019). Using insurance data for the general population, the same article also reported that during the 2nd pandemic wave, excess mortality was higher for Blacks than whites aged 1–14 years, there were no differences in mortality by race for those aged 15 to 19, and mortality was higher among whites than Blacks for those aged 20 to early 50s (Økland & Mamelund, 2019). During the third pandemic wave, mortality rates were higher in Blacks than in whites in all age groups (Økland & Mamelund, 2019). Brewer examined incidence rates at the racially segregated Camp AA (a military camp) in Humphreys, Virginia during the 2nd pandemic wave and found that white regiments experienced higher incidence rates than most Black regiments (Brewer, 1918). Frankel examined incidence rates among life insurance policy holders in the US population and discovered a similar pattern, with higher rates for whites than Blacks during the first three months of the pandemic (Frankel & Dublin, 1919). For instance, during the height of the pandemic, white women experienced rates of 1723 compared with 1522 per 10,000 among Black women (Frankel & Dublin, 1919). Lastly, Britten examined outcomes among US localities during the 1918 pandemic and found that across almost all cities, white populations experienced higher rates of influenza than Black populations, after adjustment for age and sex (Britten, 1932).

We also found thirteen studies exploring racial/ethnic disparities in the 2009 pandemic (Castrodale, 2009; Dee et al., 2011; Esteban-Vasallo et al., 2012; Kumar et al., 2012; Levy et al., 2013; Mayoral et al., 2013; Navaranjan et al., 2014; Nyland et al., 2015; Placzek & Madoff, 2014; Quinn et al., 2011; Soyemi et al., 2014; Thompson et al., 2011; Truelove et al., 2011). Black and Hispanic populations in the US had generally worse outcomes in the 2009 pandemic as compared to the white population. One study based in Illinois reported that hospitalization rates were 2–3 times greater for Black (36/100,000) and Hispanic (35/100,000) populations than white populations (13/100,000) (Soyemi et al., 2014). During the first wave of the 2009 pandemic in Wisconsin, hospitalization rates were highest among Black, Hispanic, and Asian residents, especially in Milwaukee. Black residents had lower hospitalization rates in the second wave as compared to other minoritized groups, but still higher rates than the white population (Truelove et al., 2011). Two nationwide studies in the US also found that the Hispanic population most often lacked access to health care and were at higher risk of exposure (Kumar et al., 2012; Quinn et al., 2011). Despite greater exposure, the Hispanic and American Indian/Alaska Native population was less likely to self-report Influenza-like illness than the white population in the US (Dee et al., 2011). Pediatric deaths were also highest among Hispanic residents and lowest among white residents in the US (Dee et al., 2011). In contrast, another study based in Massachusetts reported that the Hispanic population was at a significantly lower risk of ICU influenza admission than whites (Placzek & Madoff, 2014). Castrodale examined mortality rates among 10 US states, and found that during the pandemic, American Indian/Alaska Native individuals experienced a mortality rate 4 times higher than those in all other racial/ethnic groups combined (3.7/100,000 vs. 0.9/100,000) (Castrodale, 2009). A study by Quinn et al. of the US population found that Black individuals had the highest susceptibility to complications based on prevalence of chronic conditions compared to white and Hispanic individuals (Quinn et al., 2011). The study by Navaranjan et al., based in Canada, found that those who tested positive for influenza were more likely to be of East/Southeast Asian, South Asian, and Black ethnicity compared to test-negative controls (Navaranjan et al., 2014). An article based in Spain by Esteban-Vasallo that examined outcomes for native individuals and immigrants (born in Europe, North Africa, sub-Saharan Africa, Latin America, North America) during the 2009 pandemic, found that infection rates during the pandemic were lower for the immigrant population than Spanish-born individuals (2396/100,000 vs. 2796/100,000), however, the differences in severe infection rates were not significant. Additionally, ICU admission of severe cases was slightly higher for immigrants than the Spain-born population (23.8% vs. 20.4%) (Esteban-Vasallo et al., 2012). Finally, a study by Nyland et al. that examined outcomes in the United Kingdom (UK), found no significant differences between white vs. non-white individuals in length of hospital stay, severity at admission, admission delay, or mortality among admitted patients, after adjusting for confounders (Nyland et al., 2015).

Last, we found only one study comparing multiple pandemics, specifically the 1918, 1957, and 2009 pandemics in New Zealand, which analyzed mortality rates for indigenous peoples (*Māori*), as compared to people of European ancestry (Wilson et al., 2012). The *Māori* had mortality rates 7.3, 6.2, and 2.6 times higher than persons of European ancestry during the 1918, 1957, and 2009 pandemics, respectively (Wilson et al., 2012).

## 4 Discussion

In this scoping review of the literature on the evidence of health disparities in the influenza pandemics of 1918, 1957, 1968, and 2009, we describe the persistence of socioeconomic and racial/ethnic disparities throughout the four prior confirmed influenza pandemics. All reviewed articles focused on the 1918 or 2009 pandemics, except for one that also included 1957 data. Given the fact that the 1918 pandemic was one of the deadliest recorded health events in history (Centers for Disease Control and Prevention), and the recency of the 2009 pandemic, this finding is not surprising. Far fewer people died during the 1957 and 1968 pandemics compared to 1918, with 1.1 and 1 million deaths vs 50 million deaths worldwide (Centers for Disease Control and Prevention; Centers for Disease Control and Prevention–; Centers for Disease Control and Prevention). There was also less media coverage of these pandemics, as healthcare professionals at the time worried about causing public panic and fear (Honigsbaum, 2020). While the impact of the 2009 pandemic was small compared to 1918, at around 151,700–575,400 deaths globally, its recency and resulting increased data availability likely facilitated research on health disparities (Centers for Disease Control and Prevention).

Across included studies on the 2009 pandemic in the United States, minoritized individuals generally had higher incidence, mortality, or hospitalization rates than whites (Dee et al., 2011; Levy et al., 2013; Soyemi et al., 2014; Thompson et al., 2011; Truelove et al., 2011). Hispanic populations also experienced elevated risk of exposure to pandemic influenza in 2009 compared to all other groups (Kumar et al., 2012; Quinn et al., 2011). Individuals of low SES, or living in areas of low SES, also experienced worse outcomes, including higher mortality in the 1918 (Clay et al., 2019; Grantz et al., 2016; Mamelund, 2006) and 2009 pandemics (Balter et al., 2010; Kumar et al., 2012; Levy et al., 2013; Lowcock et al., 2012; Mayoral et al., 2013; Placzek & Madoff, 2014; Rutter et al., 2012; Thompson et al., 2011). One study of the 1918 pandemic did find lower mortality rates in census tracts with higher unemployment rates in Chicago. However, people of higher SES who could afford to not work were considered unemployed in the 1918 data (Grantz et al., 2016) and the highest 1918 mortality rates in Chicago were among those 21–44 years old, who made up most of the working-age population. The higher SES of some unemployed people and high mortality rates in the 21–44 age group may help explain the counterintuitive finding of higher mortality among the employed vs. unemployed (Grantz et al., 2016).

In contrast with the 2009 findings, and the recent COVID-19 pandemic (Bilal et al., 2022; Mackey et al., 2020; Shiels, Haque, & Haozous, 2021), during the 1918 pandemic, the Black population experienced lower influenza incidence and morbidity; a surprising finding given the large racial mortality disparities in the early 20th century and in subsequent pandemics. Black populations did experience higher case fatality rates than whites, which may be because of increased baseline lung disease, malnutrition, inadequate access to care, lower SES, and poor housing conditions, all stemming from structural racism (Krishnan et al., 2020). There are also several other potential explanations for the lower incidence and mortality among the Black population. First, the lower incidence among Black individuals may be due lower detection and reporting of influenza cases (Krishnan et al., 2020). A recent study examining current socioeconomic biases in seasonal influenza surveillance found that measures used for both surveillance and research may be biased against low SES individuals and racial/ethnic minorities, as they are less likely to be part of the most commonly used surveillance networks (Scarpino et al., 2020). This type of bias may have been even more pronounced in 1918, when surveillance and vital registration systems were far less developed. Second, according to Krishnan, segregation may have also prevented Black individuals from being in close contact with whites during the 1918 pandemic, reducing their risk of contracting influenza (Krishnan et al., 2020). Krishnan also hypothesizes that protective immunity from greater exposure to a less virulent early wave, as a result of living in overcrowded high exposure risk areas, potentially led to lower susceptibility among Black populations to the deadlier fall/winter wave (Krishnan et al., 2020). Brewer also suggests that the worse living conditions in tents for Black military members may have created conditions for more limited spread because of potential for less overcrowding in tents compared to buildings (Brewer, 1918). In a very recent study, Eirmann et al. tests these hypotheses (Eiermann et al., 2022), as well as potential reduced disparities from non-pharmaceutical interventions, greater implementation of protective behaviors among the Black population, and differential racial urban-rural migration and prior exposure to the 1890 influenza epidemic. They find evidence that provides suggestive support for a combination of two hypothesis: 1) that race-specific migration patterns created a differential exposure to the 1890 influenza, producing greater vulnerability among the white population, and 2) that Black population's exclusion from health care and public health systems may have encouraged greater community-based education and prevention, encouraging behaviors that reduced exposure to infections (Eiermann et al., 2022). Last, we found no studies examining disparities among the Hispanic population during the 1918 pandemic, potentially due to the small Hispanic population in the US at that time (around 1.3 million by 1920) (Gutmann, 2006) and, maybe more importantly, the lack of measurement of Hispanic ethnicity. In general, the scope of 1918 studies was smaller in size, with a number of them including single cities (or smaller samples of cities) or military populations.

During the 2009 pandemic, both SES and race/ethnicity predicted influenza incidence, hospitalization, and ICU stay (Kumar et al., 2012; Levy et al., 2013; Mayoral et al., 2013; Placzek & Madoff, 2014; Thompson et al., 2011). For example, factors that low-income minoritized populations tend to disproportionately experience such as household overcrowding, inability to engage in social distancing because of work, lack of sick leave and paid time off, and job insecurity predict influenza-like-illness incidence (Kumar et al., 2012). These same factors, as well as more limited access to health care and greater susceptibility to severe disease, because of underlying structural inequities, likely contribute to the persistent socioeconomic and racial disparities identified in the included articles. For example, neighborhood disinvestment has led to a range of structural housing conditions and employment with inadequate protections and crowded working conditions, which potentially increase the risk of exposure to the viruses that cause pandemic influenza. Additionally, non-white populations who experience overcrowding, lower household income, and lower educational attainment than their white counterparts tend to lack information on preventative measures and are at an increased risk of hospitalization (Levy et al., 2013; Mayoral et al., 2013; Thompson et al., 2011).

This scoping review has some limitations. We used a three-pronged search strategy to scope the public health field, but may have missed some articles that examined disparities (or inequalities or inequities) but did not label them as such, or articles written in languages other than English as well as those not available via standard indexing platforms. Additionally, our search was limited to the public health literature, and our initial search strategy was limited to PubMed (though our secondary searches included articles that may have been indexed elsewhere). This scoping approach may miss research indexed in other search engines, including articles from economics and history, that may examine the effects of the prior pandemics on disparities from other disciplinary perspectives or on related outcomes such as economic impacts (Clay et al., 2018; Fourie & Jayes, 2021; Garrett, 2007; Johnson & Mueller, 2002; Mourits et al., 2021; Tuckel et al., 2006). Moreover, our although the choice of a scoping review was intentional given the research question and the anticipated heterogeneity and complexity of the evidence, we are unable to provide quantitative summary measures of the association between SES and health outcomes across all pandemics. Pooling results of the studies included in this review would be inappropriate given the differences in the definitions of outcomes and other methods. (Peters et al., 2015). Future research should consider literature published in other disciplines and new literature search tools, such as elicit.org, which employ language models to automate literature searches.

Finally, we focused on health outcomes (e.g., incidence, hospitalization, ED use) and did not analyze outcomes related to vaccination or testing rates, though vaccination was not available during the 1918 pandemic. The included studies generally employed mean comparisons of health between social groups within countries, but other measures of disparities compare health outcomes across individuals, by examining the range or variance of a given measure across a population (Arcaya et al., 2015), and inferences about the direction and magnitude of disparities may depend on the disparity measure employed (Harper et al., 2010). Additionally, because our study is a scoping review rather than a meta-analysis, we qualitatively describe the direction of disparities for each pandemic, but do not present the direct estimates from each study. The SES and racial categories employed by studies varied substantially within and between pandemics (e.g., enumerator impression of household economic conditions (Sydenstricker, 1931), versus education level (Lowcock et al., 2012)) precluding direct comparisons between disparity estimates. Furthermore, we did not include formal assessment of methodological quality (Peters et al., 2015), including assessment of confounding factors, representativeness or the data, or other issues around article quality.

Since the beginning of the COVID-19 pandemic, racial/ethnic minorities and people of low SES have been disproportionately impacted by COVID-19 (Bassett et al., 2020; Chen & Krieger, 2020; Mackey et al., 2020; Bilal et al., 2021; American Public Media Research Lab Staff; Bilal et al., 2020; The COVID Racial Data Tracker). Structural factors, including structural racism, economic inequality, and segregation place Black, Latino, and Indigenous people as well as those of lower SES at greater risk of being exposed to the virus, developing severe disease if infected, and dying from the disease (Bassett et al., 2020; Chen & Krieger, 2020; Mackey et al., 2020; Bilal et al., 2021; American Public Media Research Lab Staff). Studies also show spatial clusters of high rates of COVID-19 in areas disinvested in due to decades of segregation (Bilal et al., 2021; Chen & Krieger, 2020).

In this study, we have shown that these patterns are not unique to COVID-19 and represent a continuation of a century of health disparities in influenza burden during pandemics. The medical and public health fields have both changed dramatically. The last century was marked by vast improvement in therapeutics, diagnostics, expansion of healthcare coverage to large groups of populations, including the creation of the Medicaid and Medicare programs in the US, and various other public health interventions (Bhatia et al., 2019; Centers for Disease Control and Prevention, 1999; Colgrove, 2002). The contribution of these various structural factors to disparities may have varied by pandemic. For example, in the 1918 pandemic there were few medical interventions to prevent hospitalization or death, and in fact some medical interventions may have exacerbated illness (Barry, 2005; Eiermann et al., 2022), so disparities in health care access may not have played an important role in the 1918 socioeconomic disparities but may have been critical to 2009 disparities. For example, one study found that Hispanic individuals and those of lower SES had lower risk of ILI-related ICU stay compared to their affluent white counterparts (Placzek & Madoff, 2014), which the authors partially attributed to lower access to care (Placzek & Madoff, 2014).

The repeat of disparities in the current COVID-19 pandemic suggests that part of the failed policy response to this pandemic may be a consequence of not fully comprehending the history of health disparities from prior pandemics, and failure to address social determinants of health (McCarthy et al., 2022). While the extent to which policy recommendations are appropriate based on scoping reviews is limited (Peters et al., 2015), the findings of this review suggest that public health must grapple with the likelihood that without action to address structural racism and economic inequities, disparities will likely persist in current and future pandemics. The public health field can directly act to integrate the recognition of differential risks and potential emerging inequities into pandemic preparedness and planning. However, reducing the upstream structural determinants of disparities requires change enacted through political systems and policy actors, with whom public health can influence and work.

## 5 Conclusion

The century-long patterns we described here highlight the long-lasting effects of structural racism and economic inequality that prevent the achievement of health equity. Prior literature has limited coverage of some populations, making it difficult to measure and address these problems. The current COVID-19 pandemic, in an era with better data, has both enabled better characterization and tracking of disparities but also a clearer understanding of how much more work we must do. Understanding the history of disparities in past pandemics should have informed initial and continued mitigation efforts to focus on vulnerable populations, as some researchers called for very early in the pandemic (Bailey et al., 2020), but a lack of understanding the persistence of disparities in past pandemic may have hindered public health's preparation for emerging disparities in the COVID-19 pandemic. Reducing COVID-19 disparities, and avoiding repetition of disparities in future pandemics requires a thorough grappling with the structural determinants of these disparities.

## Contributorship statement

AD and UB conceived the study. AD conducted the literature search and extracted results, supported by UB and ASM. AD wrote the first version of the manuscript, supported by ASM and UB. All authors reviewed the manuscript, provided critical content, and approved the final version.

## Funding

UB, PHM, ASM and ML were supported by the 10.13039/100000179Office of the Director of the 10.13039/100000002National Institutes of Health (NIH) under award number DP5OD26429. ML is supported by NIH/10.13039/100006545National Institute on Minority Health and Health Disparities R21MD012352-02S1. ASM was supported by 10.13039/100000060National Institute of Allergy and Infectious Diseases K01AI168579. The funding sources had no role in the analysis, writing or decision to submit the manuscript.

## Research and ethics approval

This study is a literature review and does not involve human subjects or animal participants.

## Declaration of competing interest

The authors declare no conflict of interest.

## Data Availability

No data was used for the research described in the article.
