# Corrigendum to “Health disparities in past influenza pandemics: A scoping review of the literature” [SSM – Population Health (2023) 21C 101314]

**DOI:** 10.1016/j.ssmph.2023.101516

**Published:** 2023-09-15

**Authors:** Angela D'Adamo, Alina Schnake-Mahl, Pricila H. Mullachery, Mariana Lazo, Ana V. Diez Roux, Usama Bilal

**Affiliations:** a Edward J. Bloustein School of Planning and Public Policy, Rutgers, The State University of New Jersey, New Brunswick, NJ, USA; b Department of Epidemiology, Johns Hopkins Bloomberg School of Public Health, Baltimore, MD, USA; c Urban Health Collaborative, Drexel Dornsife School of Public Health, Philadelphia, PA, USA; d Department of Health Management and Policy, Drexel Dornsife School of Public Health, Philadelphia, PA, USA; e Department of Community Health and Prevention, Drexel Dornsife School of Public Health, Philadelphia, PA, USA; f Department of Epidemiology and Biostatistics, Drexel Dornsife School of Public Health, Philadelphia, PA, USA; g Department of Health Services Administration and Policy, Temple University College of Public Health, Philadelpha, PA, USA

The authors would like to inform the readers that in the original version of the above-mentioned article we referred to “Norway” but should have referred to “Sweden,” in reference to the Bengtsson et al., 2018 study in the text and Table 1.

The authors would like to apologize for any inconvenience caused.

